# Practical Observations on the Teeth

**Published:** 1852-01

**Authors:** 


*Practical Observations on the Teeth, designed for Popular Use.* By Henry Jordan. London, Highley, pages 104, octavo.

The author has dictated his treatise to Mr. Bell, but in publishing this work for popular use, does not claim any originality, as regards discoveries in the science of dental surgery, but states that the principles he advocates and wishes to propagate in the minds of his readers, are those now generally received and acted upon by the scientific dentist, consequently the work itself is not intended for the instruction of the profession: he observes, "the scientific practitioner has nothing to gain by making a secret of the principles of his art, and can well afford to leave all mystery in connection with it, to those who live by empiricism; and whilst the public remain quite unprotected in any other way from the extravagant pretensions of the unscrupulous character, it is certainly most important that every aid should be afforded them of judging between such persons, and those who practice their profession in a just and scientific manner." In the above remarks, we most cordially agree with our author, and trust many similar works, propagating the same judicious advice, &c., will be given to the public, it will be by these means, the public will become enlightened, and protected against the gross fraud, and impositions practiced upon them.

Passing over the author's quotation, from an "elegant writer," we notice he advocates the use of the forceps for the extraction of teeth, and observes, "Although Mr. Clendon *may* not have been the first to use the *adapted* forceps, which he recommends, to him, at least, belongs the merit of having directed the attention of the profesion and the public to this subject, in such a manner, as more than anything else must have tended to the general conviction of the unsuitableness of the key, and the great superiority of properly constructed forceps, for extracting the teeth. Mr. Jordain, is evidently unacquainted with the fact, that to Mr. Tomes belongs the merit of having drawn public attention to adjusted forceps, he having published in the *Medical Gazette,* a description, with drawings, two or three years previous to the appearance of Clendon's work; we mention this fact, that in case the author should again have occasion for a reprint, we feel assured an opportunity has been given him to correct this error. He is thoroughly opposed to the use of anæsthetic agents in the extraction of teeth, and strangely enough follows in the wake of previous authors who have opposed their introduction for this purpose: he says "in the unconscious state, induced by the inhalation of these vapors, it is not always possible to get the mouth in the most favorable position, especially for extracting the grinding teeth of the lower jaw. The mouth is also very often rigidly closed, and though it may be ***generally easily*** opened, the assistance of a *second* person is often necessary to keep it so; this necessarily impedes the view, and forms an obstacle to the exact adjustment of the instrument. In the extraction of the back teeth, a half-closed state of the mouth, will often effectually prevent the proper elevation or depression of the handle of the forceps, so as to enable the operator to use it at the proper angle, and thus the power cannot be applied in the right direction, and a greater amount of force is consequently necessary to remove the tooth, and there is great danger of its being fractured.

Thus I think the use of anæsthetic agents in the extraction of teeth, tends to complicate the operation, and for the sake of the *uncertain* expectation of saving a moment's pain, a great amount of risk is incurred of rendering it unsuccessful. On these accounts, I have now entirely laid them aside, and though they may still be used by some few, I believe that dentists generally will be found to condemn their employment.

Our author and us have journeyed together, and enjoyed each other's company, up to this point. We never criticise closely or severely young authors, who write popular works for their own and the public good; simply, that if the *matter* be tolerably well sifted, a little chaff, in a *local* district, will do no harm. Upon principle, however, we must put our veto against the sweeping assertions of our author, when he says, "the use of anæsthetic agents, tends to complicate the operation of the extraction of teeth, he must surely mean that *experience* and *time*, are the essentials, not the difficulty in managing the position of the patient's head; the rigidity of the muscles, will, as a matter of course, depend upon the length of *time* and the *experience* of the operator, in administering his agent, to enable him to open the patient's mouth; he must not quarrel with the agent, he must look closer at home, and become thoroughly experienced in its administration and effects, before he ventures to condemn the greatest blessing ever given to relieve suffering humanity; experience and time will convince Mr. Jordain, that he has "popularly" treated a subject he does not at *present* understand, or *his* patients permitted to reap the benefit.

With these exceptions, we heartily recommend this work to our non-professional readers, as containing a large amount of the best advice upon the preservation of the teeth. R.

